# Field evaluation of 3-(N-acetyl-n-butyl) aminopropionic acid ethyl ester - IR3535 as a spatial repellent to control malaria: A Randomised, Before-After-Control-Intervention trial

**DOI:** 10.1371/journal.pone.0353351

**Published:** 2026-07-30

**Authors:** Joaquim Domingos Lequechane, Luís Filipe Lopes, Luzia Gonçalves, Nicole Azevedo, Ana Paula Abílio, Ana Bernardo Duajá, João Luís Manuel, Henrique Silveira

**Affiliations:** 1 Global Health and Tropical Medicine, Associate Laboratory in Translation and Innovation Towards Global Health, LA-REAL, Instituto de Higiene e Medicina Tropical, Universidade Nova de Lisboa (IHMT-NOVA), Lisbon, Portugal; 2 Centro de Investigação Operacional da Beira (CIOB), Instituto Nacional de Saúde, Ministério da Saúde, Cidade da Beira, Mozambique; 3 Centre for Ecology, Evolution and Environmental Changes (cE3c), Faculdade de Ciências da Universidade de Lisboa, Lisboa, Portugal; 4 Centro de Estatística e Aplicações da Universidade de Lisboa (CEAUL), Lisbon, Portugal; 5 z-Stat4life, Espaço Cowork Baldaya, Lisboa, Portugal; 6 Fundação Belmiro de Azevedo, Porto, Portugal; 7 Instituto Nacional de Saúde, Maputo, Mozambique; Clinton Health Access Initiative, UNITED STATES OF AMERICA

## Abstract

**Background:**

Malaria remains a major public health challenge in sub-Saharan Africa. Increasing mosquito resistance to insecticides can significantly limit current vector control strategies. Spatial repellents represent a promising complementary tool by disrupting mosquito human-seeking behaviour. This trial aimed to evaluate the efficacy of IR3535 as a spatial repellent in Tambai, a high-transmission rural area in central Mozambique.

**Methods:**

This was a randomised, Before-After-Control-Intervention trial. Four Tambai blocks were randomly selected, with 40 households randomly assigned to receive IR3535 spraying and 40 assigned to as a no-repellent control group. *Plasmodium falciparum* malaria prevalence was estimated in children under 5 years of age using polymerase chain reaction, before, at year one and at year two. Data were first analysed using frequentist methods and subsequently using spatiotemporal Bayesian models, incorporating INLA-based models to assess the impact of repellent intervention on mosquito density and diversity, sporozoite rates, and malaria prevalence in the repellent and control groups.

**Results:**

A total of 5716 mosquitoes were collected, 57.2% were *Anopheles* and 42.7% were *Culex* sp.*. An. funestus* s.l. females, were the most prevalent mosquito both indoor (92.7%) and outdoor (82.5%), followed by *An. gambiae* and *An. tenebrosus*. The total number of *An. funestus* s.l. females was lower in the repellent group compared with the control group [indoors: 765 vs 1,432; outdoors: 175 vs 293], as was the average number of mosquitoes collected per household [indoors: 18.6 vs 35.0; outdoors: 4.3 vs 7.1]. Spatiotemporal analysis revealed evidence of repellent-associated reduction in mosquito densities, with species-specific and indoor-outdoor differences, highlighting *An. funestus* s.l. dominance and spatial-temporal clustering patterns. *P. falciparum* prevalence among children under five declined from 72.6% (95% CI = 64.1–80.2) at baseline to 51.3% (95% CI = 43.5–59.1) at year one and 43.3% (95% CI = 35.7–51.1) at the end of the study, with similar reductions in both repellent and control groups. Sporozoite rates were slightly lower in the repellent group, particularly outdoors, however, these differences could not be directedly attributed to the repellent.

**Conclusion:**

Our findings indicate that the use of IR3535 as a spatial repellent reduces indoor and outdoor malaria vector densities, which may contribute to reduced malaria transmission.

**Trial registration:**

ClinicalTrials.gov NCT04419766

## Introduction

Malaria is a preventable and curable disease, nevertheless, its control and elimination remain a global challenge, particularly in sub-Saharan Africa. An estimate of 282 million cases and 610 000 deaths occurred in 2024 worldwide, with WHO African region being the most affected, accounting almost 94% of cases and 95% of malaria deaths, Mozambique being the fifth and seventh country with highest malaria cases and deaths, respectively [1]. Insecticide-treated bed nets (ITN) and indoor residual spraying (IRS) have been the main strategies for controlling the disease and have significantly reduced malaria cases and deaths from 2000 to 2015 [2]. However, the emergence of vector resistance to insecticides, combined with decreasing budgets for malaria programmes, could compromise the elimination of the disease. Additionally, ITN and IRS primarily target indoor adult mosquitos leaving residual transmission a persistent concern. This gap can be addressed by combining current control tools with innovative approaches, such as the use of spatial repellent, to achieve malaria control and its elimination by 2030 [3].

Malaria transmission to humans requires contact with an infected female mosquito seeking a blood meal. Female mosquitoes primarily locate human hosts through their olfactory system, which also influence the mosquito spectral preferences during host seeking activity [4]. The use of insect repellents that disrupt mosquito’s sense of smell can interfere with this behaviour, protecting humans from mosquito bites, reducing the risk of malaria transmission [5].

Spatial repellents are compounds containing chemical substances that keep mosquitoes away from the human host, preventing contact and mosquito’s blood-feeding, and ultimately providing protection against mosquito bites and malaria [6,7]. The use of spatial repellents is among the new and innovative strategies recommended by the WHO to address vector behaviour that impacts the effectiveness of the main malaria control interventions [8]. Laboratory assays and field trials of products with spatial repellent properties should be evaluated for their potential public health use, following the appropriate WHO guidelines [6]. Despite the existence of different repellents, some of which tested as spatial repellent, none has yet been approved by WHO.

Transfluthrin is a pyrethroid that, unlike permethrin (commonly used in ITNs), has a high vapour pressure at ambient temperature, which makes it a primary choice for use as a spatial repellent. Several studies have demonstrated its protective efficacy as a spatial repellent to reduce human‑vector contact for several vector species in different locations [7,9–13]. However, the potential impact of spatial repellents on the frequency of resistance markers [14] and the development of behavioural resistance to transfluthrin may pose a future challenge. Most recently a molecular mechanism associated with this resistance described by Okeyo *et al*. [15], highlighting the importance of continued research into alternative spatial repellents.

Several topical mosquito repellents are approved to prevent mosquito bites. These include N,N-diethyl-m-toluamide (DEET); 2-(2-hydroxyethyl)-1-piperidinecarboxylic acid 1-methylpropyl ester (KrBR 3023, Bayrepel/picaridin); Para-menthane-3,8-diol (PMD); and 3-[N-butyl-N-acetyl]-aminopropionic acid, ethyl ester (IR3535) [3]. All these compounds could potentially be repurposed for use as spatial repellents. IR3535 is a synthetic insect repellent developed by Merck in the 1970s and has been used in Europe for over 30 years [3]. It is structurally related to β-alanine (OPP Chemical Code 113509; CAS No. 52304-36-6) [15]. IR3535 can act through different mechanism, that might vary with the target organism, it can interact with odorant receptor such as CquiOR136 [16] or the GPCR receptor - mAChR [17] or Drosophila bitter-sensing receptor GR47a [18]. IR3535 is considered a masking repellent in *Anopheles coluzzii*, as it reduces attraction to humans by decreasing the amount of odorants reaching the mosquito [19]. In a recent study, a skin formulation containing 20% IR3535 reduced mosquito bites by 98%, outperforming DEET [20]. Given its properties, IR3535 is of interest as a potential spatial repellent, particularly in the context of increasing insecticide resistance to the main insecticides used to impregnate bed nets and indoor residual spraying. To date, the field efficacy of IR3535 as a spatial repellent has not been evaluated. This trial therefore aimed to evaluate the IR3535 as a spatial repellent in a high-transmission rural area in central Mozambique.

## Materials and methods

### Study settings

The study was conducted in Tambai, a remote rural community in Nhamatanda District, Sofala Province, central Mozambique, located within the Mutundo Health Center a catchment area. Tambai has a tropical climate with a rainy season from October to March and a dry season from April to September. Tambai is resource-limited setting with a high malaria burden and limited access to health services. The community comprises 12 quarters and an estimated population of approximately 1,410 inhabitants.

Housing conditions were similar in the intervention and control groups and consisted mainly of rudimentary structures built with local materials. Most houses walls were made of mud or straw/grass, packed earth floors, thatched straw roofs, and reed doors. The surrounding area is largely grass and shrub vegetation, with numerous potential mosquito breeding sites, that are partially or fully covered by vegetation, particularly during the rainy season. A forest area is located near the community. Subsistence agriculture is the main livelihood of the population.

Access to Tambai is challenging, which has historically limited the community’s access to malaria control interventions. The primary malaria control strategy implemented by the District Health Services is the distribution of long-lasting insecticide treated bed nets through antenatal care services and periodic mass distribution campaigns. The most recent mass distribution of mosquito nets to the community occurred in 2019, following the Idai cyclone. Although routine distribution through antenatal care continues, recurrent stock shortages remain a persistent in this health area.

### Study design and ethics

A randomised, Before-After-Control-Intervention trial, was conducted in Tambai. The study protocol was reviewed and approved by the Institutional Ethic Committee of the National Institute of Health from Mozambique and National Bioethics Committee for Health from Mozambique in 2020 (ref.63/CNBS/20). Written informed consent for indoor and outdoor spraying, child testing, and monthly mosquito collections was obtained from heads of households or the children’s legal guardians prior to each study procedure. For participants who could not read and write, an impartial witness was identified to attest to their consent. The study protocol was registered at ClinicalTrials.gov with the ID: NCT04419766. The trial occurred between the 1^st^ of May 2021 and 24^th^ of July 2023.

The primary outcomes of the study were: a) monthly changes in indoor and outdoor. The Secondary outcome was the community participatory mapping of mosquito breeding sites [21].

### Sample sizes and statistical power

Entomological study: The household was defined as the primary sampling unit. To account for the longitudinal nature of the entomological data and the expected correlation between repeated measures, the sample size was determined using simulations for Linear Mixed Models (LMM) via the *longpower* package in R. Considering statistical and logistical requirements, we assumed a scenario with 80 households (40 per group) followed over 24 monthly visits. The power analysis incorporated an autoregressive correlation structure (AR1) with ρ = 0.40, a total variance normalized to unit scale, and an estimated effect size (δ= 0.57) based on preliminary data from the study area. To ensure robustness under real-world conditions, the simulation accounted for a progressive attrition rate in the last six visits, assuming up to 20% missing data by the final visit. Under these assumptions, with a type I error of 5%, the study achieves a statistical power of at least 80% to detect changes in mosquito counts, considering moderate-to-large effects (S1 Fig).

Malaria in children: While the study area’s population includes approximately 111 children under five years of age (estimated at 17.5% of the 630 inhabitants) [22] all children in this age group were included in our study to maximize the available descriptive data. However, malaria prevalence is treated as a secondary exploratory outcome, as the study was primarily powered for entomological indicators.

### Pre-intervention and household selection

**Fig 1:**
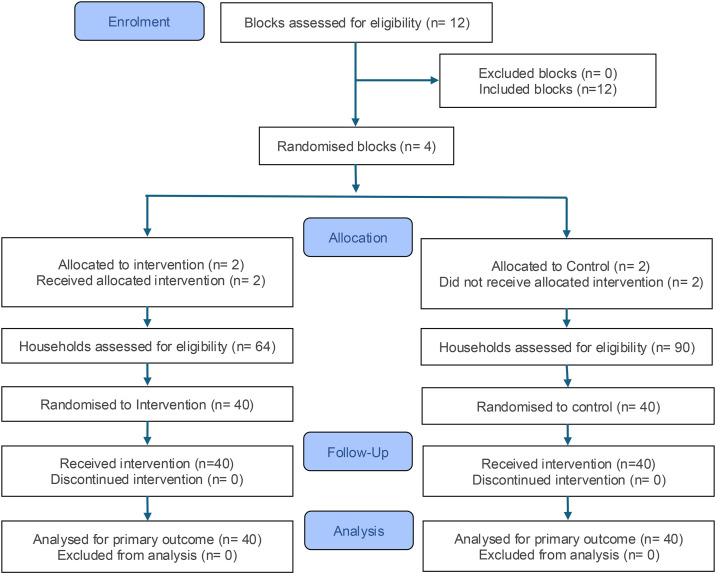
Flow diagram of the trial progress through the phases of a randomised trial of two groups: Repellent and Control. Blocks refer to community administrative blocks or neighbourhoods.

Tambai community has 12 blocks, of which four were randomly selected for the study, using simple random sampling. Two blocks assigned to the intervention group (IR3535 spraying), and two blocks assigned to the control group (without-spraying). All households in the four randomly selected blocks were mapped and georeferenced using GPS. Households with houses without one of the walls or ceilings or with inadequate structure for spraying, were excluded according to World Health Organization Guideline for malaria vector control [23]. Within each study group, 40 households were randomly selected for mosquito collection, giving a total of 80 households (40 repellent; 40 control) (Fig 1). The household was the unit of delivery. Randomization of blocks and households were performed using SPSS version 20.0.

We conducted an acceptability survey with the community and their leaders to explore their opinions about spraying indoor and outdoor of their houses with a spatial mosquito repellent prior to study implementation. All procedures and requirements for indoor and outdoor spraying by WHO were followed [24,25]. Additionally, an evaporation tank was built by the community to dispose the repellent waste according to recommended by WHO. recommendations [24]. The construction was supervised by the Sofala Province team of Mozambique’s National Malaria Control Programme [Programa Nacional de Controlo da Malária (PNCM)]. Five local community members were recruited and trained for indoor and outdoor IR3535 spraying (including storage, preparation, and waste management of the repellent) and monthly mosquito collection.

### Study procedures

#### Indoor and outdoor spraying.

**Fig 2:**
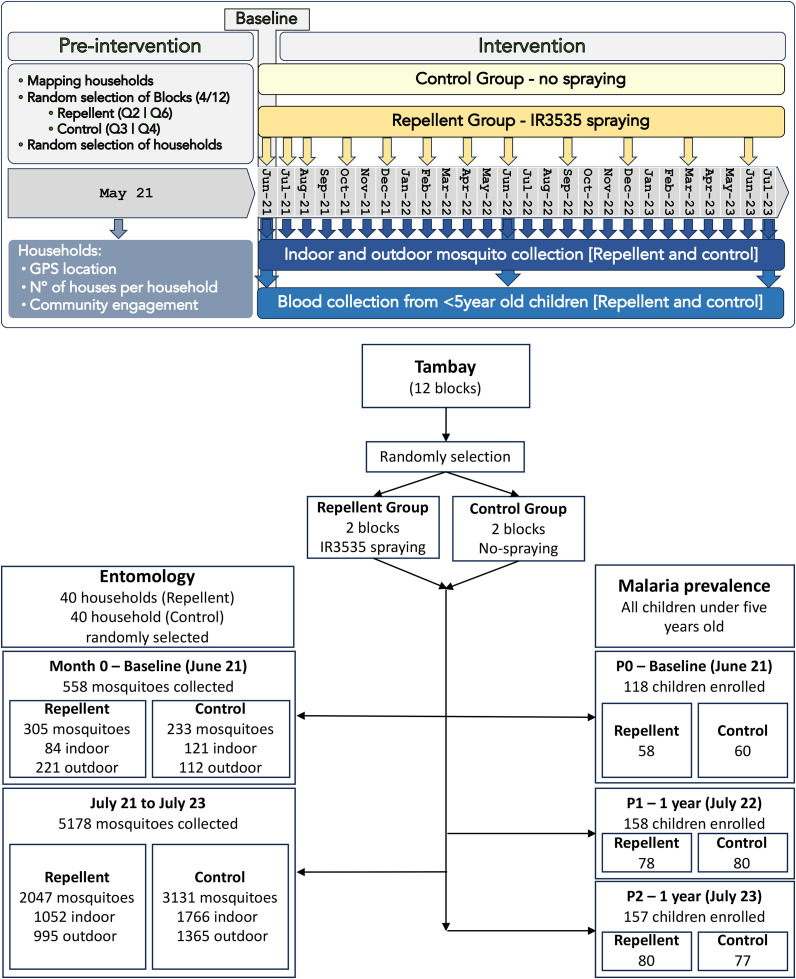
Trial procedures, timeline and profile. Block 1 to 6; P0 – malaria prevalence at baseline, P1 – malaria prevalence 1 year later, P2– malaria prevalence 2 years later (end of the project).

Indoor and outdoor spraying with IR3535 started in July 2021. Spraying was performed in all households in the two intervention blocks for the duration of the trail as described below. Twelve indoor and outdoor spraying sessions were performed early morning for 24 months in the households randomized for intervention, following the manufacture instructions (Smart Innovation, Portugal). Briefly, to each 25L of water was added 0.95g of IR3535 to spray roughly 400m^2^ of house walls. Spraying was performed monthly during the first 3 months, bimonthly over the following 6 months, and then quarterly thereafter. All households in the repellent blocks were sprayed. The indoor and outdoor spraying with IR3535 was carried out in the same households by five local project technicians supervised by the study team and PNCM team from Sofala province, according to the WHO guideline [24]. Households in the control group were not sprayed (Fig 2).

#### Mosquito collection and identification.

Mosquitoes were collected by the community project technicians trained and under supervision of project team and PNCM team from Sofala province. Mosquito collection was supported by paper-based data collection form. Adult mosquitoes were collected indoors using Prokopack aspirator (John W. Hock Company, USA) and outdoors using CDC light traps (John W. Hock Company, USA). Outdoor adult mosquitoes were collected from 19:00–07:00 using CDC light traps, and immediately after trap collection, indoor resting mosquitoes were aspirated in the same households. Baseline mosquito collections were conducted in June 2021, before the start of the intervention (IR3535 indoor and outdoor spraying). After the intervention began mosquitoes were collected monthly from July 2012 until July 2023, during 10 consecutive days each month (Fig 2). Collected mosquitoes were transported from the field to the Nhamatanda Rural Hospital Laboratory in collector cups, under cold conditions (containers with accumulators), where they were cold-anesthetized at −20°C for 10 minutes, then placed in falcon tubes containing silica gel and sent to the insectary of the NMCP in Beira, Sofala Province.

#### Malaria diagnosis.

Malaria prevalence among children under 5 years old was determined immediately before intervention [P0 (baseline)] at the end of 1st year [P1] and end of the 2^nd^ year (end of the project) [P2] (Fig 2). Blood samples were collected by 4 experienced laboratory technicians after receiving training on the study procedures. After consent for participation, a brief questionnaire was performed using Open Data Kit (ODK) based on a data collection form. Blood finger prick was collected for: i) one step malaria antigen *Plasmodium falciparum* histidine-rich protein 2 (PfHRP2) Rapid Diagnose Test (RDT) (SD Bioline Malaria Ag p.f), ii) thick and thin blood smear, used to confirm the RDT results, performed in the same day of sample collection, and iii) FTA Cards (Qiagen) for posterior DNA extraction and Polymerase Chain Reaction (PCR) analysis. To each participant was attributed a unique identification. All children diagnosed positive for malaria were treated according to Mozambique Malaria treatment guideline [25]. Children under 5 years old on malaria treatment during the study data collection period were excluded. All other Children under 5 years from all households were included.

### Laboratory procedures

#### Mosquito samples.

Mosquitoes species were identified using Gillies and DeMeillon taxonomic keys [26,27]. Mosquitoes were identified to species level, or species complex, for the *Anopheles* genus, or to the genus level for *Culex* sp. Mosquitoes. The physiological stage of female mosquitoes was determined according to the method described by Detinova (1962) [28]. Both procedures were conducted by the PNCM team from Sofala at the provincial insectary. Afterwards, mosquitoes were stored in 1.5 mL tubes containing silica gel and maintained at −20 °C until further processing. Female *Anopheles* mosquitoes were stored individually, while non-*Anopheles* species were stored in pools.

For DNA extraction, the head and upper thorax of individual mosquitoes were collected into 200 μL of sterile Phosphate Buffered Saline (PBS) and homogenized using a motorized sterile pestle for 1 minute. The homogenate was added to 5 mL of 5% Chelex 100 resin (Bio-Rad, Portugal) and incubated at 56 °C for 20 minutes. The suspension was vortexed for 10 seconds, incubated at 100 °C for 8 minutes, vortexed again, and then centrifuged at 2.0817g for 4 minutes. The supernatant was collected and stored at −20 °C.

Pools of DNA extracted from 10 *Anopheles* mosquitoes were initially screened for the presence of *Plasmodium falciparum* DNA using PCR. When a pool tested positive, individual mosquitoes were subsequently tested. The PCR assays were conducted using the *Pf_S18S* primers and probe, as previously described by [29]. The presence or absence of *P. falciparum* DNA was used to calculate the sporozoite rate, defined as the number of PCR-positive mosquitoes divided by the total number of *Anopheles* mosquitoes tested.

#### *Plasmodium falciparum* in human samples.

Thick and thin blood smears were prepared from each sample, fixed with methanol, air-dried in the field, and stained with 10% Giemsa for 15 minutes in the laboratory of Nhamatanda Rural Hospital. Parasite density was estimated by counting 200–500 white blood cells (WBC) per smear and calculated using the following formula [(no. of WBCs/no. of infected erythrocytes) × 8000 (assuming a standard WBC count of 8,000 cells/μL)]. All slides were examined by two experienced microscopists. In cases of discrepancy, a third expert microscopist provided a final reading to resolve inconsistencies.

For the molecular diagnosis, DNA was extracted from 5 mm diameter punch for each sample using Chelex 100 resin, as previously described by Lequechane et al. 2024 [30]. Detection of *P. falciparum* DNA was performed using the same PCR protocols applied to mosquito samples. Due to its higher sensitivity and specificity, subsequent analysis was performed with molecular data.

### Statistical analysis

Data were entered and curated in Microsoft Excel, then exported, coded and analysed using Statistical Package for Social Sciences (SPSS) version 29.0 and R (version 4.5.1). Categorical and continuous variables were presented as proportions, frequencies, mean and standard deviation (SD) or median and interquartile range (IQR). Chi-square (χ2) or Fisher Exact tests were used to compare malaria prevalence between repellent and control group and to compare malaria prevalence between different timepoints [baseline (P0), one year later (P1) and end of the study (P2)]. Friedman test was used to compare the monthly female *Anopheles* mosquito density indoor or outdoor in the repellent and control group. The Mann-Whitney-Wilcoxon test was used to compare female *Anopheles* mosquito density and sporozoite rate between repellent and control group. Statistical significance was defined as p < 0.05. Confidence intervals (frequentist analysis) and credibility intervals (Bayesian analysis) were obtained for parameters of interest (both are designated by 95% CI for convenience).

#### Spatiotemporal models.

Spatiotemporal Bayesian hierarchical modelling frameworkAll analyses were conducted within a Bayesian spatiotemporal hierarchical modeling framework implemented using the Integrated Nested Laplace Approximation (INLA). This framework was used consistently across entomological, malaria prevalence, and sporozoite infection models, allowing coherent inference on intervention effects while accounting for spatial and temporal dependence in the data. Spatial autocorrelation was modeled using a stochastic partial differential equation (SPDE) approach with a Matérn covariance function, representing spatially continuous Gaussian random fields. Penalized Complexity (PC) priors were specified for the spatial random effects, with priors defined as P(range<0.02)=0.01 and P(σ>1)=0.01, reflecting weakly informative assumptions on the spatial scale and marginal variability. Temporal autocorrelation was modeled using a first-order random walk (RW1), with a Gamma prior placed on the precision parameter $\tau \sim \text{Gamma}(\text{1},\text{5}\times {\text{1}\text{0}}^{-\text{5}})$.

Across all models, intervention effects were evaluated using a Difference-in-Differences (DiD) structure, including fixed effects for treatment assignment, time period (pre- vs. post-intervention), and their interaction. This interaction term represents the estimated effect of the intervention while controlling for baseline differences and temporal trends common to treated and control areas. Model adequacy and complexity were assessed using the Deviance Information Criterion (DIC) and the Watanabe–Akaike Information Criterion (WAIC), with lower values indicating improved expected predictive performance relative to model complexity. Additional diagnostic assessment was conducted using marginal log-likelihoods and Conditional Predictive Ordinates (CPO), which provide leave-one-out cross-validation–based measures of predictive adequacy. Posterior summaries were inspected to ensure numerical stability and appropriate model fit.

#### Mosquito density models.

Spatiotemporal Bayesian models were fitted to evaluate the effect of the spatial repellent IR3535 on female mosquito densities. Indoor and outdoor mosquito collections were analysed both separately and jointly, reflecting differences in collection procedures and environmental conditions. Data were aggregated by geographic location (latitude and longitude), mosquito species, collection round, treatment assignment, and collection setting (indoor or outdoor). Records lacking species identification, geographic coordinates, or key covariates were excluded.

Mosquito counts were modeled using a negative binomial likelihood with a log link to accommodate overdispersion and excess zeros. Expected counts were normalized by the number of dwellings per household using an offset on the log scale, such that regression coefficients are interpreted as effects on mosquito density per dwelling.

Let $y_{it}$ denote the mosquito count at location i and time t. The model is specified as:


$$\begin{array}{c} y_{it}\sim \text{NegBin}\left(\mu_{it},\kappa \right), \end{array}$$

$$\begin{array}{c} \text{log}(\mu_{it})=\alpha +\beta_{\text{1}}\;{\text{trat}}_{i}+\beta_{\text{2}}\;{\text{post}}_{t}+\beta_{\text{3}}\;({\text{trat}}_{i}\times {\text{post}}_{t})+\gamma^{⊤}{𝐙}_{it}+u(s_{i})+v(t)+\text{log}(E_{i}), \end{array}$$

where κ  denotes the dispersion parameter of the negative binomial distribution, controlling the degree of overdispersion relative to a Poisson model [i.e., $\text{Var}(y_{it})=\mu_{it}+\mu_{it}^{\text{2}}/\kappa$]. $E_{i}\;$ represents the number of dwellings at location i and is included as an offset term. ${𝐙}_{it}\;$ is a vector of covariates (including mosquito species, collection setting, and interaction terms), and γ  is the corresponding vector of regression coefficients. The term $u\left(s_{i}\right)\;$ represents the spatial random effect modeled using the SPDE approach, and v(t) denotes the temporal effect modeled as a first-order random walk (RW1).

#### Malaria prevalence models.

Malaria prevalence was analyzed using spatiotemporal Bayesian hierarchical models at the household level to evaluate the effect of the IR3535 spatial repellent intervention. Data were collected at three survey rounds corresponding to the pre-intervention, during-intervention, and post-intervention periods. Households were georeferenced using latitude and longitude coordinates, allowing explicit modelling of spatial dependence. The outcome variable was binary, indicating the presence or absence of malaria infection at the household level.

Let $Y_{it}\;$ denote the malaria infection status of household i at time t, where $Y_{it}=\text{1}\;$ indicates infection and $Y_{it}=\text{0}\;$ otherwise. The outcome was assumed to follow a Bernoulli distribution,


$$\begin{array}{c} Y_{it}\sim \text{Bernoulli}(p_{it}), \end{array}$$

with the probability of infection modelled using a logit link:


$$\begin{array}{c} \text{logit}\left(p_{it}\right)=\alpha +\beta_{\text{1}}\;{\text{trat}}_{i}+\beta_{\text{2}}\;{\text{post}}_{t}+\beta_{\text{3}}\;\left({\text{trat}}_{i}\times {\text{post}}_{t}\right)+\delta^{⊤}{𝐗}_{it}+u\left(s_{i}\right)+v(t). \end{array}$$

Here, ${\text{trat}}_{i}\;$ denotes treatment assignment (treated vs. control), ${\text{post}}_{t}\;$ indicates the post-intervention period, and the interaction term $\left({\text{trat}}_{i}\times {\text{post}}_{t}\right)\;$ represents the Difference-in-Differences estimate of the intervention effect. The vector ${𝐗}_{it}\;$ includes individual- and household-level covariates: sex, age, and number of children in the household. The term $u\left(s_{i}\right)\;$ represents a spatially structured random effect modelled using a stochastic partial differential equation (SPDE) approach with a Matérn covariance function, while v(t)  denotes a temporally structured random effect modelled as a first-order random walk (RW1) over survey rounds.

Model adequacy and complexity were assessed using the Deviance Information Criterion (DIC) and the Watanabe–Akaike Information Criterion (WAIC), with lower values indicating improved expected predictive performance relative to model complexity. Conditional Predictive Ordinates (CPO) were examined to assess model stability.

#### Sporozoite rate models.

Sporozoite infection status in mosquitoes was analyzed using spatiotemporal Bayesian hierarchical models to assess the effect of the IR3535 spatial repellent on mosquito infectivity. The binary outcome indicated the presence or absence of *Plasmodium falciparum* sporozoites in individual mosquitoes collected indoors and outdoors. A Difference-in-Differences (DiD) structure was adopted to estimate intervention effects while accounting for baseline differences and common temporal trends.

Let $Z_{it}$ denote the sporozoite infection status of mosquito i at time t, where $Z_{it}=\text{1}\;$ indicates infection and $Z_{it}=\text{0}\;$ otherwise. The outcome was modelled as:


$$\begin{array}{c} Z_{it}\sim \text{Bernoulli}(q_{it}), \end{array}$$

with infection probability modelled using a logit link:


$$\begin{array}{c} \text{logit}(q_{it})=\alpha +\beta_{\text{1}}\;{\text{trat}}_{i}+\beta_{\text{2}}\;{\text{post}}_{t}+\beta_{\text{3}}\;({\text{trat}}_{i}\times {\text{post}}_{t})+\eta^{⊤}{𝐖}_{it}+u(s_{i})+v(t). \end{array}$$

Here, ${\text{trat}}_{i}$ denotes treatment assignment (treated vs. control), ${\text{post}}_{t}$ indicates the post-intervention period, and the interaction term represents the DiD estimate of the intervention effect. The vector ${𝐖}_{it\;}$ includes mosquito species and collection setting (indoor vs. outdoor). The spatial random effect $u\left(s_{i}\right)\;$ was modelled using a stochastic partial differential equation (SPDE) approach with a Matérn covariance function, while temporal autocorrelation was modelled using a first-order random walk (RW1) over collection rounds.

Because treated and control areas were directly adjacent, a sensitivity analysis was conducted to assess potential boundary interference. An alternative model extended the baseline specification by including a distance-based spillover covariate, defined as an exponentially decaying function of the distance from each mosquito collection point to the nearest location in the opposite treatment arm. Spillover exposure was defined for control locations only, with an additional interaction allowing spillover effects to vary after intervention implementation.

Model adequacy and complexity were evaluated using the Deviance Information Criterion (DIC) and the Watanabe–Akaike Information Criterion (WAIC), with lower values indicating improved expected predictive performance. Conditional Predictive Ordinates (CPO) were used to assess model stability.

## Results

### Mosquito density and diversity

A total of 5716 mosquitoes (5192 ♀/ 524 *♂*) were collected, of which 57.3% [3276 (2938 ♀/ and 331 *♂*)] were *Anopheles* spp. and 42.7% [2440 (2247 ♀/ and 193 *♂*)] were *Culex* sp. (S1 Table). The majority of *Anopheles* mosquitoes were collected indoors, while the opposite occurred with the mosquitoes from the *Culex* genus. A larger number of mosquitoes was collected in the control group when compared with repellent group. Among *Anopheles* spp. mosquitoes, 1167 were collected in the repellent group 2102 in the control group, and for *Culex* sp. mosquitoes1178 were collected in the repellent group and 1262 in the control group (S1 Table).

Three *Anopheles* species were identified – 90.5% were from the *An. funestus* complex (2665♀/ 299*♂)*, 8.3% *An. gambiae* complex (241♀/ 32♂) and 0.9% *An. tenebrosus* (32♀/ 0♂) (S1 Table).

### Distribution of *Anopheles* spp. female mosquitoes’ density indoor and outdoor between repellent and control group from July 2021 to July 2023

#### Indoor.

*An. funestus* s.l. represented the majority [92.7% (2197)] of anopheline mosquitoes collected indoors followed by *An*. *gambiae* s.l. [7.0% (166)] and *An. tenebrosus* [0.3% (8)] (Tables 1 and S1). Within the *An. funestus* s.l. 65.2% (1432) were collected in the control group while 34.8% (765) were collected in the repellent group. For *An*. *gambiae* s.l., the amount collected in the repellent group and control groups was similar. Out of the 8 *An. tenebrosus,* 37.5% (3) were collected in the repellent group and 62.5% (5) were in control group. A total of 310 *Culex* sp. female mosquitoes were collected, out of which 53.2% (165) were collected in the repellent group and 46.8% (145) in the control group (Table 1).

**Table 1:** Female mosquitos collected according to species, place of collection: indoors and outdoors, and treatment group: repellent and control.

Mosquito species	Indoors			Outdoors			Control	Repellent	Total
	Control	Repellent	Total	Control	Repellent	Total			
*Anopheles funestus* ♀	1432	765	2197	293	175	468	1725	940	2665
*Anopheles gambiae* ♀	83	83	166	48	27	75	131	110	241
*Anopheles tenebrosus* ♀	5	3	8	14	10	24	19	13	32
*Anopheles* spp. ♀	1520	851	2371	355	212	567	1875	1063	2938
*Culex* spp. ♀	145	165	310	1026	911	1937	1171	1076	2247
Other non-identified ♀	0	0	0	4	3	7	4	3	7
Total no.of ♀	1665	1016	2681	1385	1126	2511	3054	2138	5192

**Fig 3:**
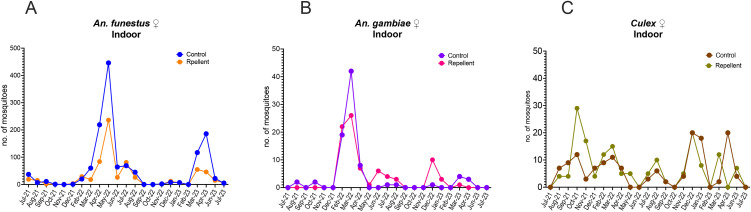
Distribution of female mosquitoes indoors during the intervention period (July 2021-july 2023). Dots represent the number of monthly collected mosquitoes. A- Monthly female An. funestus s.l. collected. B- Monthly female An. gambiae s.l. collected, and C- Monthly female Culex spp. collected.

In 2022, *An. funestus* s.l. peak collection was in May, while in 2023 the peak occurred in April. Regarding *An. gambiae*, in 2022 the peak occurred in March, while in 2023 the peak was in December 2023. The *Culex* sp. collection peak occurred in October, both in 2021 and 2022 (Fig 3 and S1 Table).

#### Outdoor.

*An. funestus* s.l., as for indoor captures, was the most represented, 82.5% (468), followed by *An*. *gambiae* s.l. [13.2% (75)] and *An. tenebrosus* [4.2% (24)]*.* Out of the 468 *An. funestus* s.l.*,* 37.4% (175) were collected in the repellent group and 62.6% (293) in the control group. For *An*. *gambiae*, 36.0% (27) were collected in the repellent group and 64.0% (48) in the control group. Out of 24 *An. tenebrosus,* 41.7% (10) were collected in the repellent group and 58.3% (14) were collected in the control group. A total of 1937 *Culex* sp. female mosquitoes were collected outdoor, where 47.0% (911) were collected in the repellent group and 53.0% (1026) in the control group. (Tables 1 and S1).

**Fig 4:**
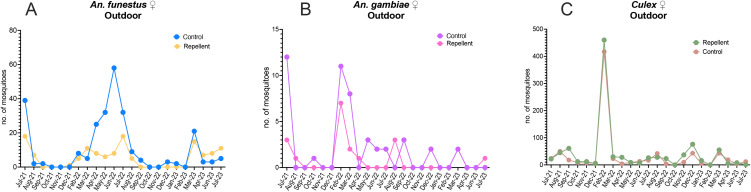
Distribution of female mosquitoes outdoors during the intervention period (July 2021-july 2023). Dots represent the number of monthly collected mosquitoes. A- Monthly female An. funestus s.l. collected. B- Monthly female An. gambiae s.l. collected, and C- Monthly female Culex spp. collected.

In 2022, *An. funestus* s.l. peak collection was in June, while in 2023 the peak occurred in March. Regarding *An. gambiae*, in 2021 the peak occurred in July, and in 2022 the peak was in February and in 2023 occurred in March. The *Culex* sp. mosquitoes peak occurred in February 2022 and March 2023. (Fig 4 and S1 Table).

The average number of *An. funestus* s.l. per household was lower in the households treated with IR3535, as the percentage of households where no mosquito was collected during the intervention (Table 2).

**Table 2:** Mosquitoes collected per household.

	*Culex* spp.		*An. funestus*		*An.gambiae*	
	averge*	%**	averge*	%**	averge*	%**
Indoors^#^						
Control	3.5	97.6	35	95.1	2.0	68.3
Repellent	4.0	97.6	18.6	90.2	2.0	65.9
Outdoors^§^						
Control	25	73.2	7.1	90.2	1.2	53.7
Repellent	22.2	73.2	4.3	75.6	0.7	43.9

*Average number of mosquitoes per household; **Percentage of household where mosquitoes were captured; ^#^ Indoor mosquitoes were captured using Prokopack aspirator; § Outdoor mosquitoes were captured CDC light traps (John W. Hock Company, USA).

### Spatiotemporal analysis of entomological data

The spillover-adjusted spatiotemporal model indicates evidence of a reduction in mosquito density in treated areas following the intervention. The treatment–time interaction was negative (posterior mean = −0.487; 95% CrI: −0.961 to −0.014), indicating that mosquito density declined after intervention in repellent areas relative to controls.

Species composition differed substantially, with *An. gambiae* s.l. and *An. tenebrosus* showing lower densities relative to *An. funestus* s.l., while *Culex* sp. exhibited more moderate reductions. Indoor collections showed higher predicted densities than outdoor collections.

**Fig 5:**
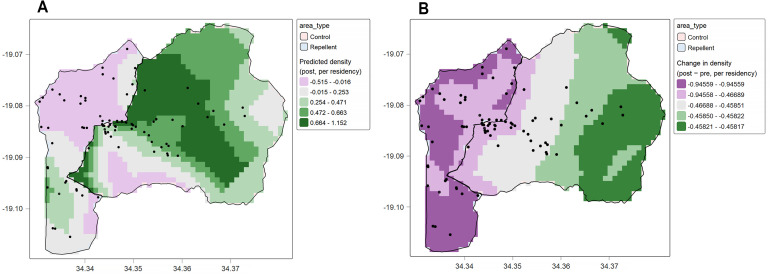
Spatial distribution of predicted mosquito density and change after intervention from the spillover-adjusted spatiotemporal model. (A) Posterior mean predicted mosquito density per residency in the post-intervention period. (B) Posterior mean change in predicted mosquito density between post- and pre-intervention periods (post–pre). Surfaces were derived from a Bayesian spatiotemporal negative binomial model including treatment assignment, time, species, indoor/outdoor location, spillover covariates, and spatial (SPDE) and temporal (RW1) random effects. Shaded polygons indicate control and repellent study areas, and points represent household locations. Colours indicate posterior mean predicted density or change, with cooler colours representing lower values and warmer colours higher values.

Spatial predictions revealed clear heterogeneity across the study region (Fig 5).

The post-intervention density surface (Fig 5A) showed localized areas of higher predicted mosquito density, particularly clustered within parts of the control zone and along the boundary between treated and untreated areas. The change surface (Fig 5B) indicated that reductions were spatially structured rather than uniform. Areas within the treated zone generally showed decreases in predicted density, while some locations near the treatment boundary showed smaller reductions or localized increases.

The spillover gradient parameters did not provide strong evidence for a consistent cross-boundary effect (post-intervention distance-weighted spillover gradient in control areas), suggesting that any spillover from treated to untreated areas was limited or spatially heterogeneous.

#### Species-specific and interaction effects.

*An. funestus* s.l. remained the predominant species group captured in both indoor and outdoor collections. All other taxa — *An. gambiae*, *Culex*, and *An. tenebrosus* — were less abundant relative to *An. funestus* s.l.. Among these, *An. tenebrosus* showed the lowest relative abundance (posterior mean = −1.496; 95% CrI: −2.457, −0.536).

Species-specific interaction terms suggested heterogeneity in response to the intervention. Relative to *An. funestus* s.l., reductions in mosquito density were somewhat weaker for *An. gambiae* (interaction mean = 0.327; 95% CrI: −0.093, 0.747) and for *Culex* (mean = 0.369; 95% CrI: 0.097, 0.641). Although uncertainty remained substantial for most interactions, the direction of effects was consistent with species-specific differences in intervention response.

The interaction between treatment effect and collection setting (indoor vs. outdoor) showed little evidence of a strong differential effect (mean = 0.065; 95% CrI: −0.200, 0.329), suggesting that the intervention’s influence on mosquito density was broadly similar across indoor and outdoor environments.

#### Mosquito behaviour.

The fed/pregnant ratio was compared between repellent and control groups. The indoor *An. funestus* s.l. fed/pregnant ratio was 4.3 for control group and 3.7 for repellent group. The indoor *An. gambiae* fed/pregnant ratio was 0.3 for control group and 0.4 for repellent group. Showing that the two species have different resting habits, despite slight changes the ratio was not significantly altered between repellent and control groups, suggesting that they were not affected by the repellent (S1 Table).

### Malaria prevalence

In the baseline survey (P0), a total of 118 children under 5 years old participated in the study. Of these, 56.8% (67) were female. The median age was 29.0 months (IQR: 17.0–40.0). Nearly half, 46.6% (55) belonged to 12–36 age group and 39.0% (46) reported having had malaria 30 days before the study visit. One year later (P1), a total of 158 children under 5 years old participated in the study and the majority 52.5% (83) was female. The median age was 19.5 months (IQR: 7.3–40.3), 39.1% (61) belonged to 12–36 age group and 42.4% (67) reported having had malaria 30 days prior to the study visit. At the end of the study (P2), 157 children under 5 years were included, of which 59.2% (93) were male. The median age was 31.0 months (IQR: 22.0–48.0), with 43.1% (66) belonging to 37–59 age group. Around 59.2% (93) of participant reported having had fever 30 days prior to the study visit (Table 3).

**Table 3:** Characterization of children tested for Plasmodium falciparum in the three different timepoints in repellent and control.

Variable	Category	P0						P1						P2					
		Repellent		Control		Overall		Repellent		Control		Overall		Repellent		Control		Overall	
		n	%	n	%	n	%	n	%	n	%	n	%	n	%	n	%	n	%
Gender																			
	Male	25	43.1	26	43.3	51	43.2	31	39.7	44	55.0	75	47.5	46	57.5	47	61.0	93	59.2
	Female	33	56.9	34	56.7	67	56.8	47	60.3	36	45.0	83	52.5	34	42.5	30	39.0	64	40.8
Age in months																			
	[IQR(Q1-Q75)]	29.0(17–40)						19.5(7.25 - 40.25)						31(22 −48)					
Age group (months)																			
	<12	10	17.2	13	21.7	23	19.5	24	31.2	27	34.2	51	32.7	12	15.0	10	13.7	22	14.4
	dez-36	26	44.8	29	48.3	55	46.6	32	41.6	29	36.7	61	39.1	39	48.8	26	35.6	65	42.5
	37 - 59	22	37.9	18	30.0	40	33.9	21	27.3	23	29.1	44	28.2	29	36.3	37	50.7	66	43.1
Fever last 2 weeks																			
	Yes	30	51.7	33	55.0	63	53.4	49	62.8	52	65.0	101	63.9	59	73.8	55	71.4	114	72.6
	No	26	44.8	27	45.0	53	44.9	29	37.2	28	35.0	57	36.1	21	26.3	21	27.3	42	26.8
	Dont remember	2	3.4	0	0.0	2	1.7	0	0.0	0	0.0	0	0.0	0	0.0	1	1.3	1	0.6
Last time head Malaria																			
	≤30 days	18	31.0	28	46.7	46	39.0	37	47.4	30	37.5	67	42.4	50	62.5	43	45.6	93	59.2
	>30 days	9	15.5	2	3.3	11	9.3	31	39.7	35	43.8	66	41.8	9	11.3	5	6.5	14	8.9
	Dont remember	27	46.6	25	41.7	52	44.1	9	11.5	14	17.5	23	14.6	10	12.5	20	26.0	30	19.1
	Never	4	6.9	5	8.3	9	7.6	1	1.3	1	1.3	2	1.3	11	13.8	7	9.1	18	11.5
	No Answer	0	0.0	0	0.0	0	0.0	0	0.0	0	0.0	0	0.0	0	0.0	2	2.6	2	1.3
Antimarial treatment received																			
	ACT	24	41.4	32	53.3	56	47.5	55	70.5	52	65.0	107	67.7	45	56.3	35	45.5	80	51.0
	Quinino							0	0.0	1	1.3	1	0.6	1	1.3	0	0.0	1	0.6
	No treatment	11	19.0	14	23.3	25	21.2	21	26.9	24	30.0	45	28.5	1	1.3	4	5.2	5	3.2
	Dont know	23	39.7	14	23.3	37	31.4	2	2.6	3	3.8	5	3.2	21	26.3	22	28.6	43	27.4
	No Answer	0	0.0	0	0.0	0	0.0	0	0.0	0	0.0	0	0.0	12	15.0	16	20.8	28	17.8

IQR – Interquartile range; Q – Quartile; P0 – Baseline data collection; P1 – 1 year later data collection; P2 – 2 years later (end of the project) data collection.

To estimate the malaria prevalence for comparing repellent and control group, only the PCR data were considered. The malaria prevalence was 72.6% (95% CI = 64.1–80.2) at P0, 51.3% (95% CI = 43.5–59.1) at P1 and 43.3% (95% CI = 35.7–51.1) at P2. There were no differences in malaria prevalence between repellent and control group at P0 (p = 0.407, χ² = 1.000), P1 (p = 0.262, χ² = 1.642) and P2 (p = 0.748, χ² = 0.154). There was a significant reduction in malaria prevalence at P1 (p = 0.005) and P2 (p = 0.036) compared to P0 (S1 Table). All children who tested positive for malaria in the community were treated with Artemisinin based combination therapy, according to National guideline for malaria case management.

**Fig 6:**
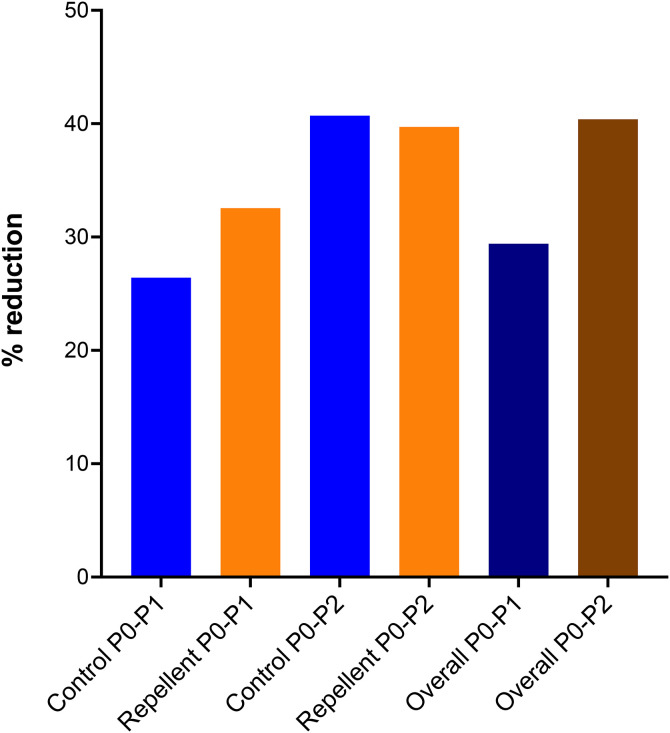
Reduction of malaria prevalence by Plasmodium falciparum between timepoint in repellent and control group. P0 – malaria prevalence at baseline, P1 – malaria prevalence 1 year later, P2– malaria prevalence 2 years later (end of the project).

Overall, there was a 29.3% reduction of malaria prevalence at P1 and 40.4% reduction at P2 related to P0. The reduction rate in malaria prevalence was 32.5% in the repellent group and 29.9% in control group at P1 compared to P0. When compared malaria prevalence from P0 to P2, the reduction was 39.6% in the repellent group and 40.7% in the control group (Fig 6).

#### Spatiotemporal analysis of malaria prevalence.

Malaria prevalence was also evaluated using a spatiotemporal Bayesian hierarchical model incorporating treatment assignment, time period, individual-level covariates, and household structure. The best-supported model included treatment group, post-intervention period, sex, age, and number of children in the household, along with spatial and temporal random effects. The post-intervention period was associated with a substantial reduction in malaria prevalence (posterior mean = −1.244; 95% credible interval: −1.938, −0.549), indicating an overall temporal decline in malaria risk across the study area. However, the interaction term representing the intervention effect (treated × post-intervention) showed no evidence of a treatment-specific impact on malaria prevalence (posterior mean = 0.165; 95% credible interval: −0.789, 1.119), suggesting that the observed reduction was not strongly attributable to the repellent intervention itself.

Sex showed a borderline association with malaria risk, with females tending to have lower odds of infection compared to males (posterior mean = −0.379; 95% credible interval: −0.787, 0.029). Age was positively associated with malaria prevalence (posterior mean = 0.015; 95% credible interval: 0.003, 0.027), indicating increasing odds of infection with increasing age. Importantly, the number of children in the household was negatively associated with malaria prevalence (posterior mean = −0.404; 95% credible interval: −0.715, −0.094), suggesting a protective association in households with more children, potentially reflecting differences in household structure or care-seeking behaviours.

This model achieved the lowest DIC (558.88) and WAIC (559.48) among the candidate models, representing a substantial improvement over simpler specifications (ΔDIC = −10.07), and supporting the inclusion of household-level covariates alongside spatiotemporal random effects.

### Mosquito infection

**Fig 7:**
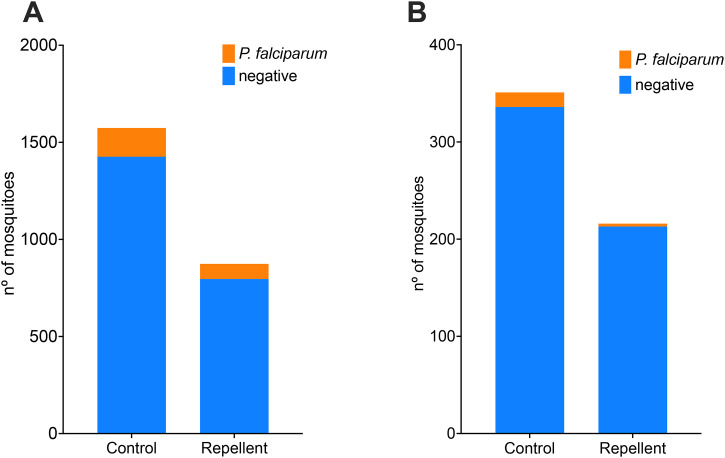
Proportion of Plasmodium falciparum sporozoite-positive Anopheles spp. mosquitoes. (A) Indoor collected mosquitoes, (B) outdoor collected mosquitoes.

The sporozoite rate observed on repellent and control group for the indoor collected *Anopheles* spp. was 9.4% and 8.9% respectively (Fig 7A), while for outdoor mosquitoes the sporozoite rates was 4.3 for control and 1.3 for repellent group (Fig 7B), when Chi-square (χ2) test was used to compare control and repellent groups, differences were not significant.

#### Spatiotemporal analysis of sporozoite infection rates.

The spatiotemporal INLA model indicated a clear overall decline in sporozoite infection probability over time (post effect: mean −1.86, 95% CrI −3.38 to −0.40), suggesting that transmission intensity decreased during the study period. Baseline differences between treatment arms were modest and uncertain, and the treatment–time interaction suggested that reductions in infection probability were not substantially stronger in repellent areas than in control areas.

A strong spatial spillover effect was detected. Control households located near treated areas showed markedly different infection dynamics after the intervention period, indicating that proximity to treated zones influenced infection risk. This effect was substantially larger than other fixed effects in the model, highlighting the importance of spatial context.

**Fig 8:**
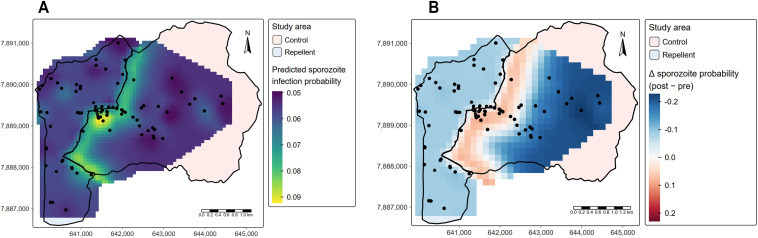
Spatial distribution of predicted Anopheles funestus sporozoite infection probability (indoor collections, spillover-adjusted). (A) Posterior mean predicted probability of sporozoite infection in the post-intervention period. (B) Predicted change in infection probability between post- and pre-intervention periods (post − pre). Surfaces represent model-based predictions from the spatiotemporal INLA model including spatial random effects and treatment–time interactions. Black points indicate household sampling locations. Study areas are outlined, with control areas shown in pale pink and repellent areas in light blue. In panel B, blue colours indicate reductions in predicted infection probability after the intervention, whereas red colours indicate increases.

The spatial random field captured pronounced local heterogeneity in infection risk, consistent with clustering at neighbourhood scales. This pattern is reflected in the predicted probability surface (Fig 8A), where areas of higher infection probability form localized hotspots rather than aligning with treatment boundaries. The change surface (Fig 8B) further indicates that reductions in infection probability were spatially uneven, with declines concentrated in some zones and stable or increased risk in others.

## Discussion

The distribution of insecticide-treated bed nets (ITNs) through mass campaigns targeting the general population, and pregnant women during antenatal care, combined with indoor residual spraying (IRS), represent the cornerstone and most effective strategies for malaria control. Despite the significant progress achieved by using these measures, residual malaria transmission continues to pose a substantial challenge to National Malaria Control Programmes, undermining efforts toward disease elimination. Our study contributes to possible complementary alternatives to current control measures, by showing that mosquitoes were mainly caught in the control group rather than in the repellent group, showing that the IR3535 used as spatial repellent reduces mosquito density. Which is in line with another study using a different repellent and a randomized time-series in Tanzania, Ulanga District, that found a reduction in the local predominant vector, *An. arabiensis* [31]. In addition, we found that the majority of *Anopheles* mosquitoes were captured indoors and predominantly within the control group, as in other a community-based interventional study investigating the use of spatial repellents, which reported a higher density of *An. funestus* indoors [9]. Such consistencies underscore the persistent indoor presence of malaria vectors, and the usefulness of spatial repellent, highlighting the ongoing need to address indoor transmission dynamics in control efforts. Even so, a limitation of this study is the mosquito collection methods used outdoors. Different sampling tools are suited to different objectives [32] and our choices may have influenced observed mosquito abundance and species composition. Although logistically and ethically challenging, all-night human landing catches would have provided a more informative proxy for outdoor transmission.

*An. funestus* s.l. was the most prevalent species in the study area, followed by *An. gambiae* s.l., which is consistent with the reported distribution of Anopheline mosquitoes in rural areas of Mozambique, where *An. funestus* s.l. was also the dominant species, followed by *An. gambiae* [33–35] confirming that *An. funestus* s.l. remains the main malaria vector in Mozambique.

A previous study evaluating DEET, IR3535, and Picaridin as insect repellents reported that IR3535 and Picaridin had no effect on *Anopheles coluzzii*, *Aedes aegypti*, and *Culex quinquefasciatus* [4], highlighting that different mosquito species may respond differently to various repellents. In contrast, our study found that female *Culex* sp. were less affected by the repellent indoors and effective on reducing in both indoor and outdoor *An. funestus* s.l. These different outcomes concerning indoor and outdoor mosquito species may be explained by variations in the mosquito species tested and the specific active ingredients in the repellents used.

A methodological approach similar to ours has been successfully applied in previous studies test the repellent action of Pyriproxyfen [36], informative [37]. Even so, our findings showed evidence that the use of IR3535 as a spatial repellent had a notable effect on density reduction of *An. funestus* s.l., the main malaria vector in Mozambique.

The spatiotemporal analysis indicates that the intervention was associated with a spatially structured reduction in mosquito density, consistent with a localized effect of the repellent rather than a uniform regional shift. The model outputs and spatial predictions indicate that the intervention likely reduced mosquito density locally, but that its effectiveness varied across space, highlighting the importance of accounting for spatial structure when evaluating vector control strategies.

Regarding the sporozoite infection, results indicate that probability declined over time, suggesting a general reduction in transmission intensity during the study period. However, the absence of a strong treatment–time interaction implies that this decline cannot be attributed solely to the intervention. The model highlights the importance of spatial processes in shaping transmission patterns. A spillover effect suggests that proximity to treated areas influenced infection dynamics, consistent with the possibility that the intervention altered mosquito movement or host-seeking behaviour, thereby redistributing transmission risk across treatment boundaries. Together, the model results and spatial predictions indicate that while transmission declined overall, the intervention’s effect was spatially heterogeneous and mediated by local ecological processes rather than producing a uniform reduction in infection risk.

Despite the reduction in malaria prevalence after one year and after two years of intervention, this reduction observed between repellent and control group, could not be attributed to IR3535 treatment. Nevertheless, the overall reduction in malaria prevalence in the community over the study period highlights the importance of continuous sensitization of the population to adopt individual and collective measures to prevent malaria transmission. This is particularly important when mosquito nets cannot be used to prevent mosquito bites and the region has high malaria prevalences, as also described in the recent demographic health survey carried out in Mozambique, which found a prevalence of 32.0%.

The primary vector control strategy employed at scale in Mozambique is the use of insecticide-treated mosquito nets (ITNs), which are distributed predominantly through mass distribution campaigns and as antenatal care (ANC) services for pregnant women by the NMCP. According to the most recent Demographic and Health Survey (DHS), approximately 57% of households own at least one ITN, which is far from the WHO recommendations for universal access [8]. In addition, reductions in financial support for malaria control programmes may significantly undermine efforts toward disease control and its elimination. Within this context, our findings show the potential of IR3535 as spatial repellent and could represent a novel and appropriate vector control tool to be incorporated in the National Malaria Control Programme, having the potential to mitigate residual malaria transmission. Further studies using more robust approaches in different settings are needed to evaluate the effect IR3535 as spatial repellent in malaria incidence.

Some limitations should be considered when interpreting the findings of this study. Although the randomized design strengthens comparability between groups, the absence of a wide physical buffer between treated and control households means that some cross-boundary influence cannot be fully excluded, despite accounting for spatial structure and spillover effects in the models. In addition, the sample size was primarily determined to detect entomological effects, and therefore the study was not powered to detect modest differences in malaria prevalence.

## Conclusions

IR3535, applied as a spatial repellent through regular indoor and outdoor spraying, reduced the density of *An. funestus* s.l., the primary malaria vector in rural Mozambique. Showing potential as a complementary vector control tool. Our results indicate that the IR3535 as spatial repellent has potential to reduce malaria vector density indoors and outdoors and could be used by National Malaria Control Programmes, especially in regions without coverage of indoor spraying and in malaria pre-elimination regions. Future controlled trials with broader geographic scope are warranted to confirm the protective effect of IR3535 on malaria transmission.

## Supporting information

S1 Fig Power curve. (PNG)

S1 Table Distribution of mosquitos collected during study implementation between years 2021–2023. (XLSX)

S1 Checklist CONSORT checklist. (DOCX)
