# Supplementary figures and images for: Field evaluation of 3-(N-acetyl-n-butyl) aminopropionic acid ethyl ester - IR3535 as a spatial repellent to control malaria: A Randomised, Before-After-Control-Intervention trial
Source: PLoS One. 2026 Jul 30;21(7):e0353351. doi: 10.1371/journal.pone.0353351 (PMC13422834; doi:10.1371/journal.pone.0353351)

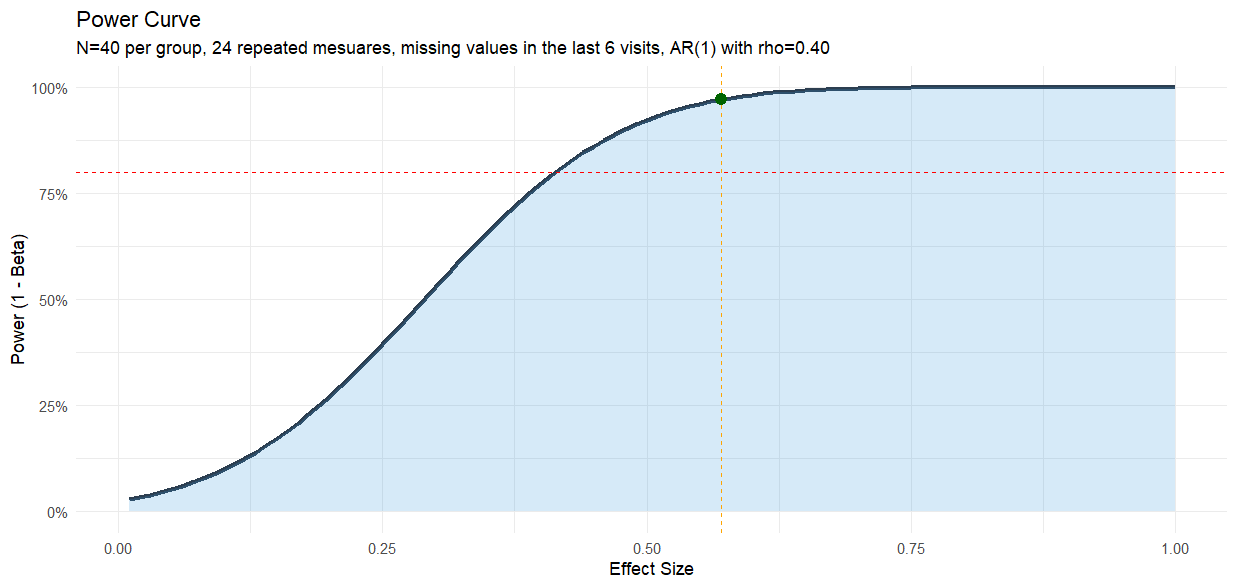

Supplement: S1 Fig — (PNG) [file pone.0353351.s001.png]
